# Case Report: Reiterating the importance of tissue biopsy in the diagnosis of tuberculosis: lessons from a case of pediatric pulmonary tuberculosis complicated by Hodgkin's lymphoma

**DOI:** 10.3389/fped.2024.1454657

**Published:** 2024-10-03

**Authors:** Yonghan Luo, Xiaotao Yang, Jun Zhou, Shuangqiong Pu, Yanchun Wang

**Affiliations:** ^1^ Second Department of Infectious Disease, Kunming Children’s Hospital, Kunming, Yunnan, China; ^2^ Faculty of Life Science and Technology, Kunming University of Science and Technology, Kunming, Yunnan, China; ^3^ Department of Pathology, Kunming Children’s Hospital, Kunming, Yunnan, China

**Keywords:** tissue biopsy, tuberculosis, Hodgkin's lymphoma, children, diagnosis

## Abstract

The clinical symptoms of tuberculosis infection in children are not typical and need to be distinguished from many diseases, and the tumor is one of them. We present a case of cervical lymphadenopathy in a child with positive purified protein derivative (PPD) and Interferon-Gamma Release Assay results, ultimately diagnosed with Hodgkin's lymphoma via cervical lymph node biopsy. We learned some lessons from the case: First, Pathological biopsy remains the “gold standard” for diagnosing tuberculosis. Second, there are limitations of sampling in lump fine needle aspiration biopsy, surgical methods for lymph node are preferred to obtain larger tissues and improve tuberculosis detection sensitivity.

## Introduction

The clinical presentation of tuberculosis infection in children is often atypical, relying heavily on laboratory test results (1, 2). Among these, Pathological findings serve as the gold standard but require anesthesia and invasive procedures (1, 3). The combination of purified protein derivative (PPD) with Interferon-Gamma Release Assay, due to its high sensitivity and specificity, is commonly employed in clinical auxiliary tuberculosis diagnosis (4, 5). However, the absence of a gold standard diagnosis may lead to misdiagnosis (6, 7). Herein, we present a case of cervical lymphadenopathy in a child with positive PPD and Interferon-Gamma Release Assay results, ultimately diagnosed with Hodgkin's lymphoma via cervical lymph node biopsy.

## Case

**Figure 1:**
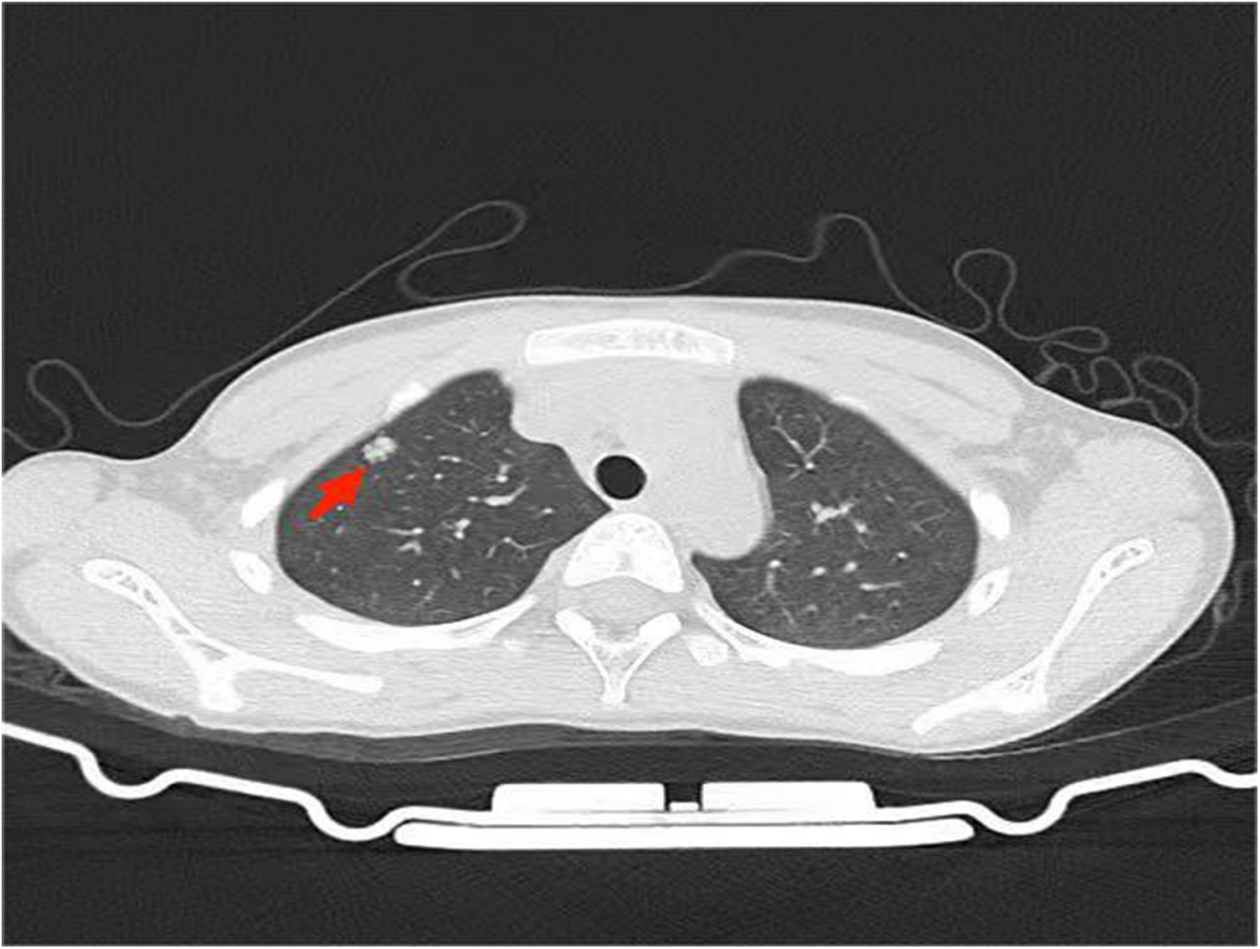
Chest computed ntomography reveals a high-attenuated lesion in the upper lobe of the right lung, as indicated by red arrows.

**Figure 2:**
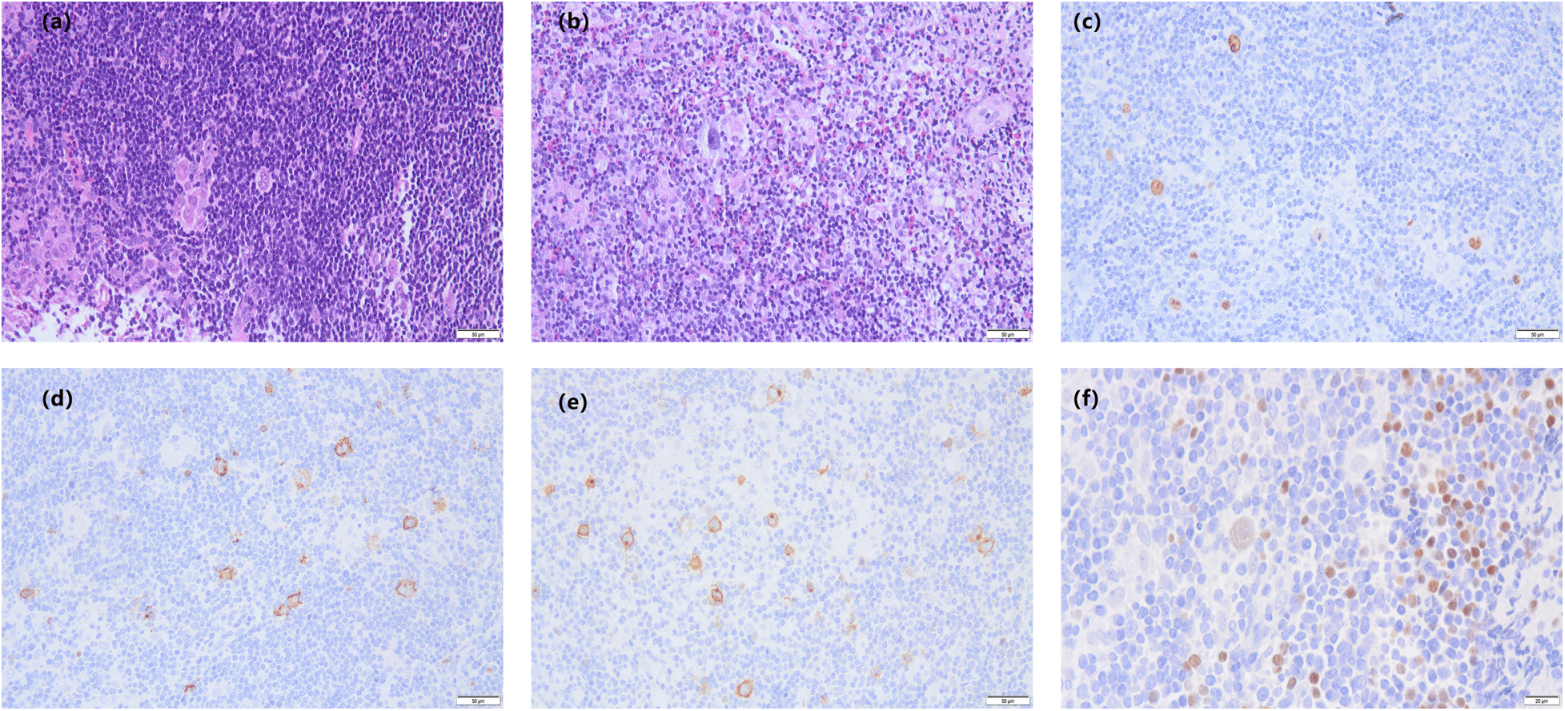
Pathological biopsy of a cervical lymph node shows classical mixed-cellularity hodgkin's lymphoma. **(a)** multiple large Hodgkin cells are observed, with prominent eosinophilic nucleoli, some resembling “mirror image” cells(HE 200×). **(b)** tumor cells are visible in the background of eosinophils and neutrophils(HE 200×). **(c)** EBER(+) *in situ* hybridization(200×). **(d–f)** show the immunohistochemical staining. **(d)** CD15(+)(200×). **(e)** CD30(+)(200×). **(f)** PAX-5(+)(400×).

An 8-year-old male child was admitted to hospital with a chief complaint of “ a neck lump for more than 3 months”. The child had a total of 3 times of fever in the course of the disease and was treated in the local hospital, where a cervical lump fine needle aspiration biopsy suggested reactive hyperplasia. Despite oral cephalosporin therapy and other medications, there was no improvement in the cervical lump. Physical examination revealed significant enlargement of the left cervical lymph nodes, approximately 4 × 2 cm in size. Interferon-Gamma Release Assay yielded a positive result, while PPD indicated a strong positive result. Chest-enhanced CT revealed nodular lesions in the right upper lung lobe, as depicted in Figure 1. Given the positive result of PPD and Interferon-Gamma Release Assay, combined with the patient's neighbor having a history of active pulmonary tuberculosis, the patient was suspected of having pulmonary tuberculosis complicated by cervical lymph node tuberculosis. Anti-tuberculosis treatment was planned accordingly. Subsequently, completion of cervical lymph node biopsy was performed to further elucidate the etiology. Pathological examination of the cervical lymph node biopsy revealed classical mixed-cellularity Hodgkin's lymphoma, as shown in Figure 2. PET-CT examination was conducted to determine lymphoma staging, indicating lymphoma in the left neck and tuberculosis infection in the nodule of the right upper lobe. Final diagnosis: (1) Cervical Hodgkin's lymphoma; (2) Tuberculous infection.

## Discussion

Initially, the patient did not undergo a lymph node biopsy or PET-CT examination. Despite the absence of symptoms such as low-grade fever, night sweats, or fatigue. CT imaging of the lungs revealed a high-density lesion in the right upper lung. Considering this site is the most common site of tuberculosis manifestation, and the treatment of conventional antibiotics in other hospitals is ineffective. There are no acid-fast bacilli in sputum, but PPD and Interferon-Gamma Release Assay are positive. Combined with his neighbor's history of active pulmonary tuberculosis, the clinical diagnosis of tuberculosis infection is clear. Therefore, it was naturally assumed that both the lung nodules and cervical lumps were attributable to tuberculous infection. However, several aspects of tuberculosis diagnosis and treatment in this patient puzzled us: First, considering that tuberculosis infection generally progresses from the lungs to the entire body. If the patient already had extrapulmonary tuberculosis (lymph node tuberculosis), indicating a prolonged duration of infection, why were there no symptoms of tuberculosis? Although tuberculosis intoxication symptoms in pediatric tuberculosis are atypical, they still warrant suspicion. Second, as the pulmonary lesions in the patient were consistent with tuberculosis imaging features, the likelihood of tuberculosis infection was higher. However, assuming that the cervical lump was also due to tuberculosis infection, why did the previous hospital's cervical fine needle aspiration biopsy only suggest reactive hyperplasia without caseous necrosis? Third, the severity of pulmonary tuberculosis and extrapulmonary tuberculosis differs, leading to variations in the types and durations of anti-tuberculosis drugs used. Fourth, histopathological biopsy remains the gold standard for diagnosing tuberculosis. Considering these reasons, we ultimately decided to perform cervical lymph node biopsy on the patient. The biopsy request encountered some resistance, as many doctors questioned why a biopsy was necessary when a tuberculosis diagnosis seemed definitive. However, the biopsy was ultimately performed after careful consideration. As is well known, aside from subtype, tumor staging plays a crucial role in determining prognosis (8). Imagine if the patient had been treated solely for tuberculosis as a matter of course, the pulmonary tuberculosis lesion would likely have been controlled, but the cervical Hodgkin's lymphoma would have inevitably metastasized throughout the body. When anti-tuberculosis treatment fails to achieve the desired effect or when the patient exhibits symptoms of other tumor metastasis, conducting a biopsy diagnosis at that time may result in lymphoma staging reaching an uncontrollable stage. Therefore, this case provides us with some insights: First, Pathological biopsy remains the “gold standard” for diagnosing tuberculosis. Second, there are limitations of sampling in lump fine needle aspiration biopsy, surgical methods for lymph node are preferred to obtain larger tissues and improve tuberculosis detection sensitivity.

## Data Availability

The original contributions presented in the study are included in the article/Supplementary Material, further inquiries can be directed to the corresponding author.
